# Oxidized high-density lipoprotein promotes CD36 palmitoylation and increases lipid uptake in macrophages

**DOI:** 10.1016/j.jbc.2022.102000

**Published:** 2022-04-29

**Authors:** Yun Zhang, Doudou Dong, Xiaoting Xu, Hui He, Yuan Zhu, Tingwen Lei, Hailong Ou

**Affiliations:** Department of Biochemistry and Molecular Biology, School of Basic Medical Science, Guizhou Medical University, Guiyang, Guizhou, China

**Keywords:** oxHDL, CD36, palmitoylation, lipid uptake, DHHC6, 2-BP, 2-bromopalmitate, ABE, acyl-biotin exchange, Cav-1, caveolin-1, CD36, cluster of differentiation 36, CHX, cycloheximide, DHHC, Asp-His-His-Cys, DiI, 1,1′-diotadecyl-3,3,3′,3′-tetramethylindocarbocyanine perchlorate, DKO, double KO, ER, endoplasmic reticulum, FA, fatty acid, HAM, hydroxylamine, HDL, high-density lipoprotein, HFD, high-fat diet, JNK, c-Jun N-terminal kinase, LDL, low-density lipoprotein, M-β-CD, methyl β-cyclodextrin, mCD36, mutant CD36, NEM, *N*-ethylmaleimide, oxHDL, oxidized high-density lipoprotein, PAT, palmitoyl acyltransferase, RCT, reverse cholesterol transport, RIPA, radioimmunoprecipitation assay, SelK, selenoprotein K, SR, scavenger receptor, wCD36, wildtype CD36

## Abstract

Oxidized high-density lipoprotein (oxHDL) reduces the ability of cells to mediate reverse cholesterol transport and also shows atherogenic properties. Palmitoylation of cluster of differentiation 36 (CD36), an important receptor mediating lipoprotein uptake, is required for fatty acid endocytosis. However, the relationship between oxHDL and CD36 has not been described in mechanistic detail. Here, we demonstrate using acyl-biotin exchange analysis that oxHDL activates CD36 by increasing CD36 palmitoylation, which promotes efficient uptake in macrophages. This modification increased CD36 incorporation into plasma lipid rafts and activated downstream signaling mediators, such as Lyn, Fyn, and c-Jun N-terminal kinase, which elicited enhanced oxHDL uptake and foam cell formation. Furthermore, blocking CD36 palmitoylation with the pharmacological inhibitor 2-bromopalmitate decreased cell surface translocation and lowered oxHDL uptake in oxHDL-treated macrophages. We verified these results by transfecting oxHDL-induced macrophages with vectors expressing wildtype or mutant CD36 (mCD36) in which the cytoplasmic palmitoylated cysteine residues were replaced. We show that cells containing mCD36 exhibited less palmitoylated CD36, disrupted plasma membrane trafficking, and reduced protein stability. Moreover, in ApoE^−/−^CD36^−/−^ mice, lipid accumulation at the aortic root in mice receiving the mCD36 vector was decreased, suggesting that CD36 palmitoylation is responsible for lipid uptake *in vivo*. Finally, our data indicated that palmitoylation of CD36 was dependent on DHHC6 (Asp-His-His-Cys) acyltransferase and its cofactor selenoprotein K, which increased the CD36/caveolin-1 interaction and membrane targeting in cells exposed to oxHDL. Altogether, our study uncovers a causal link between oxHDL and CD36 palmitoylation and provides insight into foam cell formation and atherogenesis.

High-density lipoprotein (HDL) facilitates cholesterol efflux and promotes reverse cholesterol transport (RCT) to the liver for metabolism, which reduces the excess cholesterol from peripheral tissues. In addition to its role in RCT, HDL has other beneficial functions in antiatherosclerosis, such as antioxidation, anti-inflammation, and endothelium-dependent vasodilatation (1). However, HDL is constantly remodeled and highly susceptible to various modifications, specifically oxidation, which is oxidized even more rapidly than low-density lipoprotein (LDL) (2, 3). Oxidized HDL (oxHDL) loses its antiatherosclerotic function, turning into a dysfunctional form, which increases macrophage lipid loading (4), impairs endothelial function (5), and exhibits proatherogenic properties (6). OxHDL level is elevated in patients with cardiovascular diseases (7). The oxHDL is detected to locate in the intima of atheromatous plaques in human abdominal aortae and found relevance to acute myocardial infarction (8, 9). Therefore, oxHDL has been shown to correlate clinically with an increased risk for cardiovascular events (10).

Cluster of differentiation 36 (CD36), a class B scavenger receptor (SR) family member, is an integral membrane glycoprotein containing 472 amino acids (11). The receptor comprises two transmembrane domains, a large extracellular domain, and two short cytoplasmic domains formed by -NH_2_ and -COOH terminal segments (11). CD36 undergoes severe modification after translation, including glycosylation, phosphorylation, ubiquitination, and palmitoylation (11). The extracellular loop contains three phosphorylation sites and multiple N-linked glycosylation sites; the C terminus at the intracellular tail has two ubiquitination sites (11). In addition, there are four palmitoylation sites on cysteine residues, two each at the extreme NH_2_ (Cys3 and Cys7) and COOH termini (Cys464 and Cys466) (12, 13).

CD36 is expressed in various cells and tissues, including macrophages, platelets, endothelial cells, adipocytes, and skeletal muscle, and is recognized by multiple ligands (14). Apart from its well-established role in facilitating long-chain fatty acid (FA) uptake, another important function of CD36 is to bind oxidized low-density lipoprotein (oxLDL) *via* oxidized phospholipids present within the particles and then endocytose it into macrophages, which promotes foam cell formation and accumulation of cholesterol in atherosclerotic plaques (14). CD36 functions as an oxHDL receptor, which mediates oxHDL uptake in macrophages and induces apoptosis in endothelial progenitor cells (4, 5). Studies have also revealed that CD36 plays a critical role in the oxHDL-triggered inflammatory response (15), which suggests that CD36 is involved in the effects of dysfunctional HDL.

Palmitoylation (S-palmitoylation), the most common form of S-acylation, is a post-translational modification characterized by covalent attachment of palmitic acid, a 16-carbon saturated FA (16:0), to cysteine residues *via* a labile thioester linkage (16). Unlike other forms of protein lipidations, such as prenylation and myristoylation, palmitoylation is a type of reversible modification (16). Palmitoylation is dynamically catalyzed by various palmitoyl acyltransferases (PATs) containing DHHC (Asp-His-His-Cys) motif and depalmitoylation by thioesterases, such as palmitoyl protein thioesterases or acyl-protein thioesterase (16). Palmitoylation increases the hydrophobicity of the target protein and affects protein membrane association, trafficking, stability, and protein–protein interactions (16).

The palmitoylation of CD36 broadly impacts physiological and pathological processes. CD36 without palmitoylation modification is retained at the endoplasmic reticulum (ER) and fails to reach the medial Golgi for further processing, which reduces the efficiency of membrane raft incorporation (17). Dynamic palmitoylation of CD36 regulates CD36-mediated FA uptake and lipid droplet growth in adipocytes (18). Increased CD36 palmitoylation and its plasma membrane localization are reported to have positive relationships with steatohepatitis (19). These previous studies on the functions of CD36 palmitoylation were mainly focused on inflammation and FA uptake (18, 19). On the other hand, the intracellular signaling pathway induced by oxHDL and molecular mechanism for the atherogenic oxHDL are much less clear as compared with oxLDL. Considering that oxHDL triggers atherosclerosis requiring CD36 as a receptor (4), we aimed to determine the effects of oxHDL on CD36 palmitoylation and investigate the role of such modification on CD36 intracellular translocation, foam cell formation, and lipid accumulation in cultured macrophages and mouse aortae.

## Results

### OxHDL promotes CD36 palmitoylation and plasma membrane translocation

**Figure 1:**
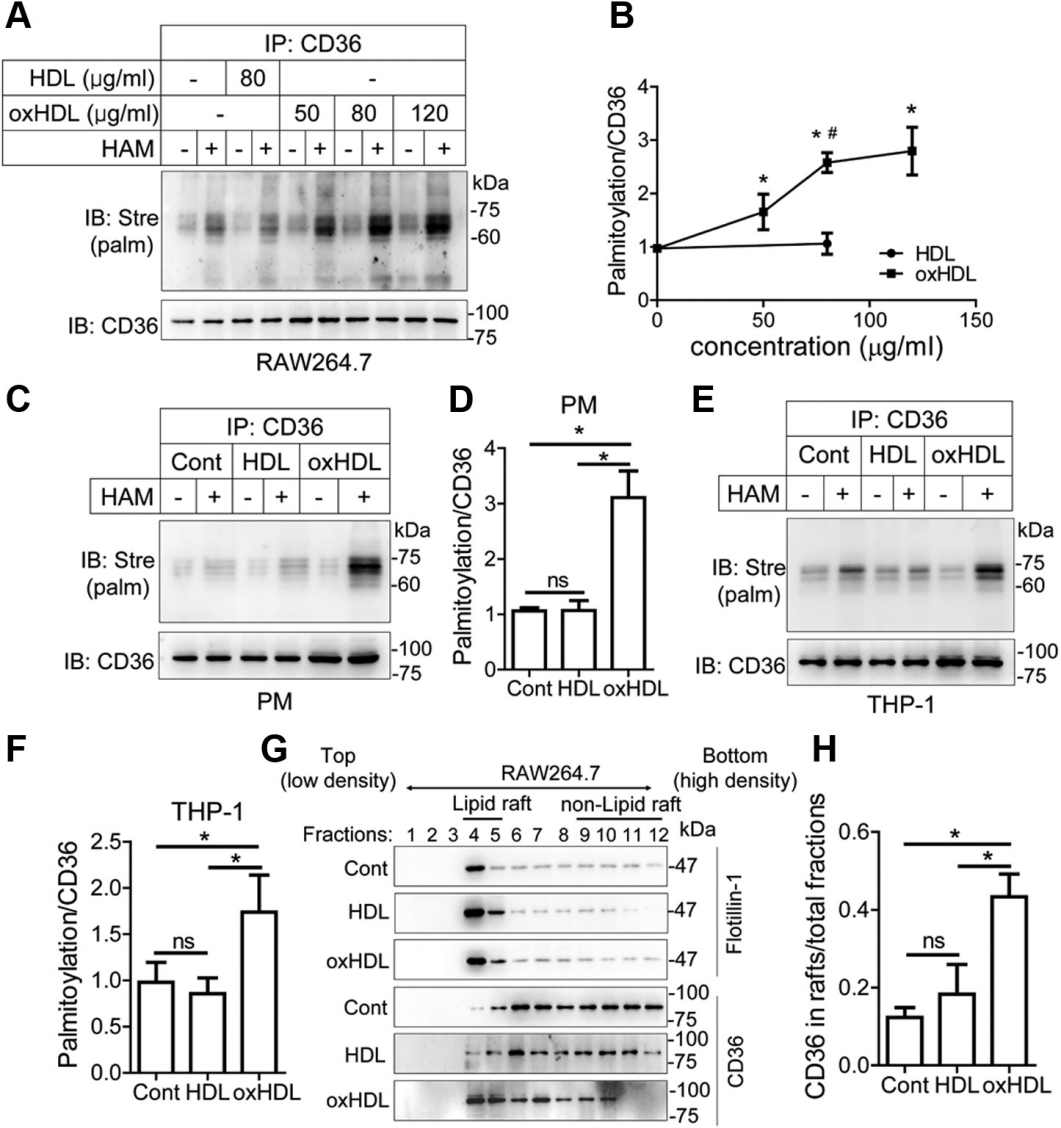
**oxHDL increases cellular palmitoylated CD36 and promotes lipid raft recruitment in macrophages.***A*, RAW264.7 cells were incubated with HDL (80 μg/ml) and various concentrations of oxHDL (50, 80, and 120 μg/ml) for 24 h. Palmitoylation modification was measured by ABE assay. ∗*p* < 0.05, compared with untreated controls. ^#^*p* < 0.05, compared with HDL (80 μg/ml). *B*, quantification of the palmitoylation in *A*. The palmitoylation level was normalized to CD36. *C*–*F*, CD36 palmitoylation in isolated peritoneal macrophages (*C* and *D*) and human THP-1 cells (*E* and *F*). The two lines of macrophages were treated with HDL and oxHDL at a concentration of 80 μg/ml for 24 h and subjected to an ABE assay. *G* and *H*, incorporation of CD36 into membrane lipid rafts in RAW264.7 cells. Cells were treated with 80 μg/ml of HDL or oxHDL. Lipid rafts recognized as detergent-resistant membranes (DRMs) were isolated by discontinuous sucrose gradient ultracentrifugation. Fractions 4 and 5 were identified as DRMs as indicated by the lipid raft marker flotillin-1. The lipid raft recruitment of CD36 was quantified by calculating the ratio of CD36 in DRMs to the CD36 in total fractions. All data were from three independent experiments, and values were expressed as mean ± SD. ∗*p* < 0.05. ABE, acyl-biotin exchange; CD36, cluster of differentiation 36; HDL, high-density lipoprotein; ns, not significant; oxHDL, oxidized high-density lipoprotein.

The prepared oxHDL was characterized, and we found that the Cu^2+^-induced oxHDL dramatically increased lipoprotein aggregation (Fig. S1*A*). The oxidized modification caused a significant reduction in cell viability treatment for 24 h when the concentration was elevated to 120 μg/ml in RAW264.7 macrophages (Fig. S1*B*). Consistently, TUNEL assays revealed that the oxHDL (80 μg/ml) showed slight cytotoxicity, whereas oxHDL at 120 μg/ml strongly triggered cell apoptosis at 24 h after treatment (Fig. S1*C*). To determine the effects of oxHDL on CD36 palmitoylation, the RAW264.7 cells were exposed to HDL and various doses of the oxHDL for 24 h. We performed an acyl-biotin exchange (ABE) assay on proteins extracted from the cells. Steadily increased CD36 palmitoylation was observed in the cells treated with oxHDL at concentrations ranging from 50 to 120 μg/ml (Fig. 1*A*). Quantitative data revealed that oxHDL at 50 μg/ml significantly induced palmitoylation modification compared with the untreated cells (Fig. 1*B*). Moreover, the palmitoylation level in cells stimulated with 80 μg/ml oxHDL was over twofold higher than cells with 80 μg/ml HDL treatment (Fig. 1*B*). We confirmed the results by treatment with HDL or oxHDL in isolated primary murine peritoneal macrophages and human THP-1 cells. Similarly, oxHDL incubation obviously increased CD36 palmitoylation compared with untreated control or HDL treatment in both types of cells (Fig. 1, *C*–*F*). Despite the increase in oxHDL, the palmitoylation level detected in HDL-treated cells was nearly the same as the level in untreated RAW264.7 cells and peritoneal macrophages and slightly reduced in THP-1 cells (Fig. 1, *A*–*F*). We also noted that the CD36 expression level was comparable in untreated and HDL-treated cells but increased when treated with oxHDL in the three cell lines (Fig. 1, *A*–*F*).

To investigate the membrane binding of palmitoylated CD36, RAW264.7 cells were treated with HDL or oxHDL, and lipid rafts, membrane microdomains with high content of cholesterol and glycosphingolipids, were separated by discontinuous sucrose density-gradient (5–40%) centrifugation. The membrane rafts identified as detergent-resistant membranes were detected in fractions 4 and 5, as evidenced by the presence of its marker flotillin-1 (Fig. 1*G*). CD36 was distributed mainly in fractions of 6 to 12 in the HDL-treated cells as well as the untreated control (Fig. 1*G*). In contrast, increased amounts of CD36 in oxHDL-stimulated cells were found in fractions 4 and 5 of the lipid raft region, suggesting that oxHDL promotes CD36 subcellular trafficking and membrane anchoring (Fig. 1, *G* and *H*).

To further investigate the impacts of oxHDL on CD36 surface expression, we performed costaining of CD36 and caveolin-1 (Cav-1), another membrane lipid raft marker, and the localization was detected by confocal microscopy. CD36-positive staining was distinct from Cav-1 in control and HDL-treated RAW264.7 cells, whereas colocalization of the two molecules was clearly observed in cells treated with oxHDL, suggesting an increase in membrane recruitment of CD36 (Fig. S2*A*). In contrast, positive signal overlap between CD36 and calnexin, an ER marker, was frequently detected in control and HDL-treated cells, indicating that CD36 tended to localize at the ER. However, the positive staining for CD36 was more often separated from calnexin in oxHDL-stimulated cells (Fig. S2*B*). The results provide direct evidence that enhanced palmitoylation promotes CD36 movement from subcellular organelles to the plasma membrane.

### OxHDL promotes the assembly of CD36 with Fyn and Lyn and activates downstream c-Jun N-terminal kinase

**Figure 2:**
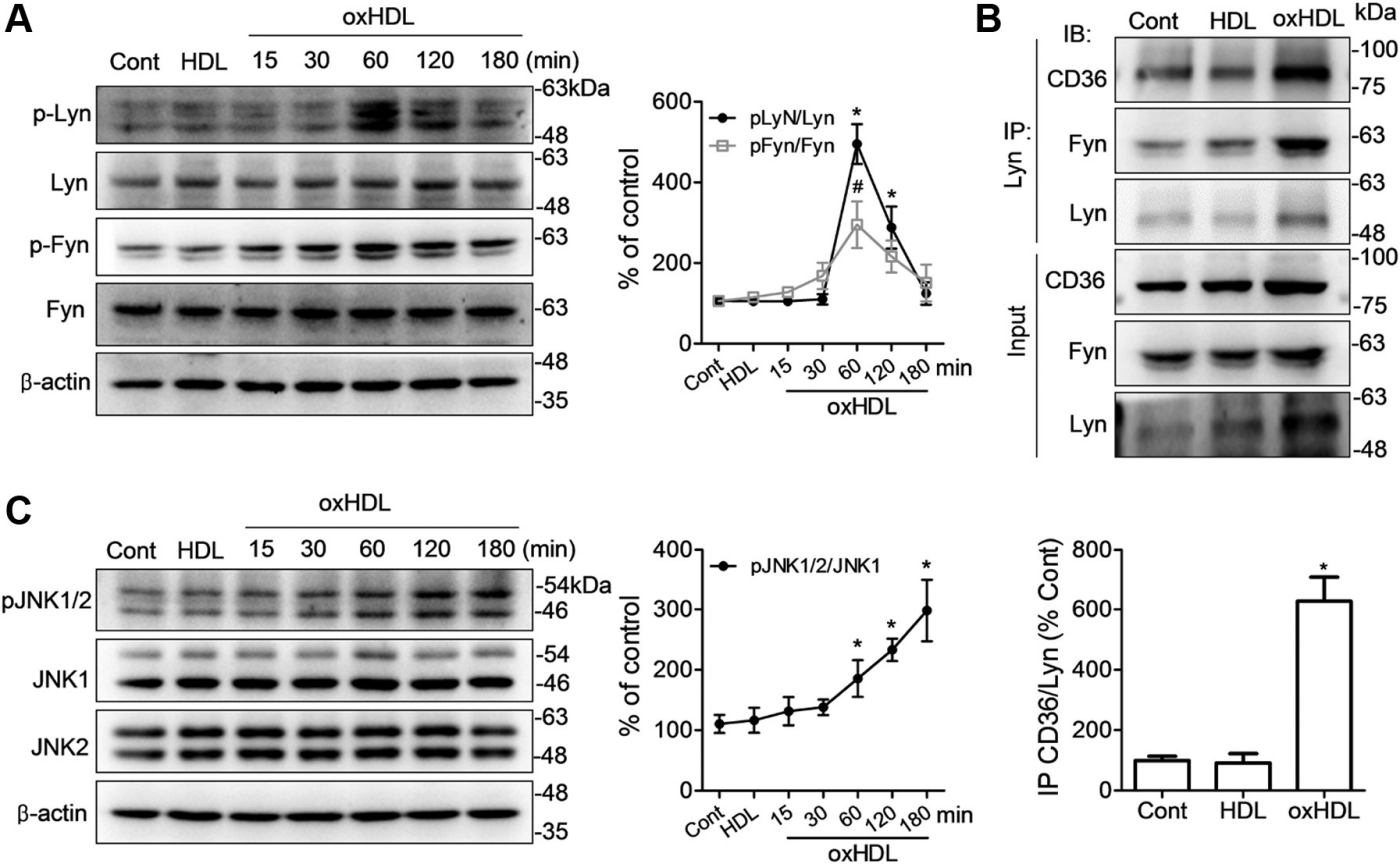
**oxHDL activates the downstream mediators Lyn, Fyn, and JNK.***A*, RAW264.7 cells were treated with HDL or oxHDL at 80 μg/ml. The incubation time is 1 h and as indicated, respectively, for HDL and oxHDL. Lysates were immunoblotted with antibodies against phospho-Lyn (Tyr508), phospho-Fyn (Tyr530), Lyn, and Fyn. Quantitative analysis of changes in the phosphorylation levels of Lyn and Fyn. Values are expressed as p-Lyn/Lyn or p-Fyn/Fyn relative to controls. *B*, CD36 assembles with Lyn and Fyn with respect to oxHDL. RAW264.7 cells were incubated with HDL and oxHDL at 80 μg/ml for 24 h. oxHDL-treated cells were immunoprecipitated with CD36 antibody and immunoblotted with Lyn, Fyn, and CD36 antibodies. *C*, Western blotting analysis of JNK phosphorylation in cells stimulated with 80 μg/ml oxHDL for different periods as indicated. Membranes were stained with antibodies against phospho-JNK1/2 (Thr183 + Tyr185), JNK1, and JNK2. The data were presented as means ± SD of three independent experiments. ∗*p* < 0.05, ^#^*p* < 0.05, compared with controls. CD36, cluster of differentiation 36; HDL, high-density lipoprotein; JNK, c-Jun N-terminal kinase; oxHDL, oxidized high-density lipoprotein.

Previous reports have demonstrated that the Src family kinases Lyn and Fyn form a complex with CD36, and the association with the kinases is required for CD36-dependent activation of the c-Jun N-terminal kinase (JNK) in foam cell and thrombus formation (20, 21, 22, 23). Therefore, we investigated whether oxHDL promotes Lyn and Fyn recruitment to CD36 and the downstream signaling cascade. RAW264.7 cells were incubated with HDL and oxHDL, and phosphorylated Fyn (p-Fyn) and phosphorylated Lyn (p-Lyn) were detected within the first 3 h. Lyn was remarkably phosphorylated (Tyr508) at 1 h after oxHDL treatment. The phosphorylation levels of Fyn at Tyr530 gradually increased from 15 to 60 min in oxHDL-exposed cells, although less notably than the phosphorylation levels detected in p-Lyn at 60 min (Fig. 2*A*). To examine the association of CD36 with Lyn and Fyn, the cell lysates were immunoprecipitated with antiLyn antibody. CD36 and Fyn levels detected by immunobloting were increased in oxHDL-treated cells compared with the untreated or HDL-treated cells, which suggests that CD36 forms a complex with Lyn and Fyn in macrophages in response to oxHDL (Fig. 2*B*).

We noted that oxHDL increased total cellular CD36, Fyn, and Lyn in our experiment in Figures 1, *A*–*F* and 2*B*. To clarify whether the increase in the detected CD36 is caused by mRNA upregulation or CD36 cellular translocation, we incubated the RAW264.7 cells with oxHDL and methyl β-cyclodextrin (M-β-CD), a lipid raft inhibitor. Both CD36 mRNA levels and protein contents were dramatically increased in oxHDL-treated cells, and the costimulation of cells with oxHDL and M-β-CD compared was with the untreated cells (Fig. S3, *A* and *B*). Moreover, there is no significant difference in CD36 mRNA and protein between cells treated with oxHDL and oxHDL plus M-β-CD (Fig. S3, *A* and *B*). We also found that oxHDL increased mRNA abundance in Fyn and Lyn (Fig. S3, *C* and *D*). Therefore, these results suggested that oxHDL induced CD36, Fyn, and Lyn transcriptions and led to increase in the protein levels.

We further tested whether oxHDL affected JNK activity. RAW264.7 macrophages were incubated with oxHDL for different times ranging from 0 to 3 h, and the phosphorylation status of JNK in the whole cell lysates was detected by immunoblotting at various time points. As expected, we observed that oxHDL treatment increased the level of p-JNK1/2 (Thr183 + Tyr185) in RAW264.7 cells at 1 h after treatment and further increased in the following 2 h (Fig. 2*C*). These data demonstrate that the downstream signaling molecule JNK is activated in response to oxHDL stimulation. Therefore, we conclude that oxHDL particles activate CD36 signaling, as evident by increasing CD36/Lyn/Fyn assembly and downstream JNK activity.

### OxHDL increases macrophage lipid uptake and promotes foam cell formation

**Figure 3:**
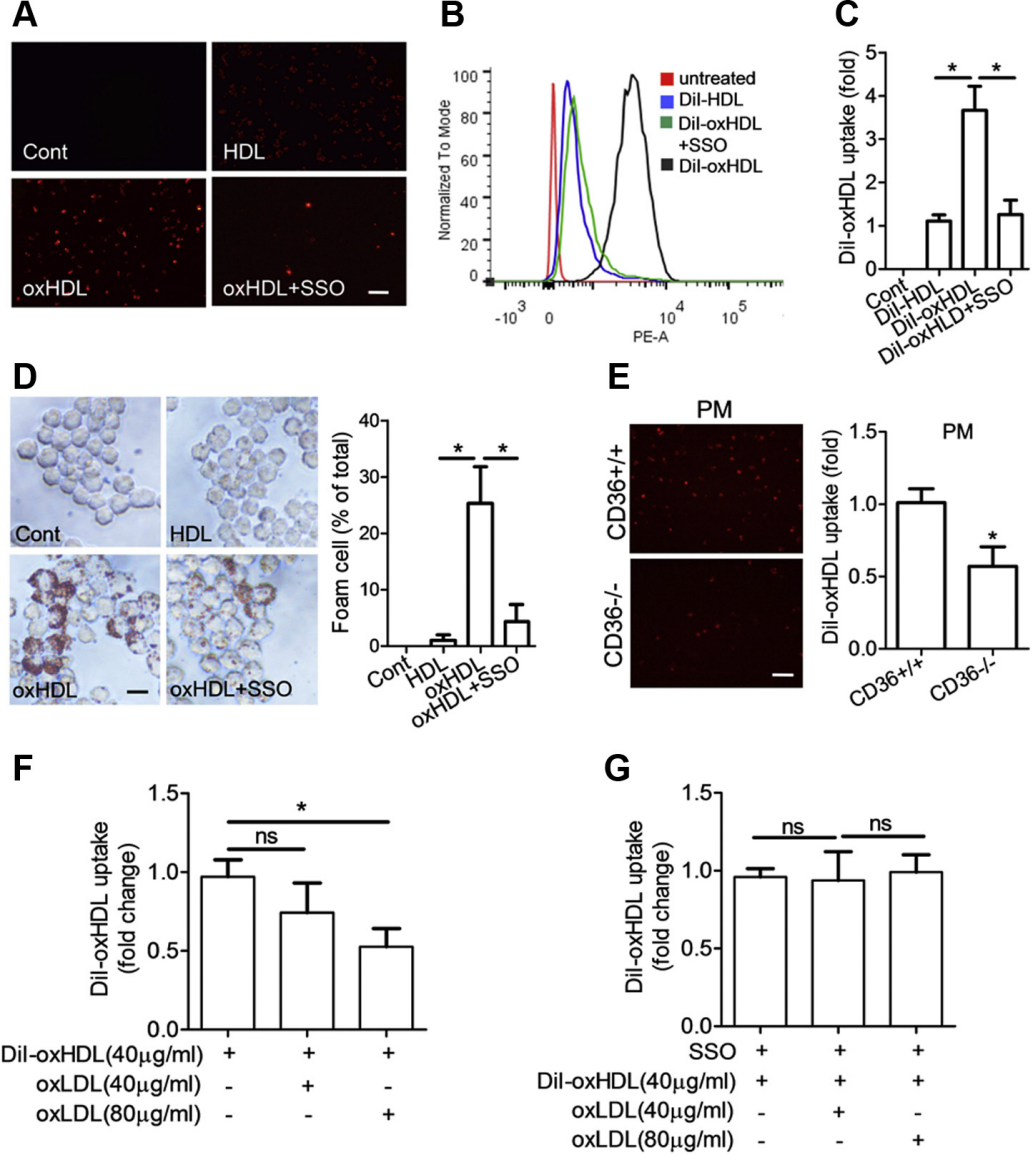
**Lipid uptake and foam cell formation in macrophages.***A*, fluorescent images for macrophage lipid uptake. RAW264.7 cells were incubated with DiI-labeled HDL (30 μg/ml), DiI-oxHDL (30 μg/ml), or DiI-oxHDL + sulfosuccinimidyl oleate (SSO) (200 μmo/l) for 6 h. The scale bar represents 50 μm. *B* and *C*, lipid uptake measured by flow cytometer. The levels are expressed as the fold change of the mean fluorescence intensity (MFI) to DiI-HDL. ∗*p* < 0.05. *D*, cellular lipid accumulation in macrophages. RAW264.7 cells were incubated with HDL (80 μg/ml), oxHDL (80 μg/ml), or oxHDL + SSO (200 μmo/l) for 24 h and stained with Oil Red O. Positive staining was analyzed by light microscopy. The scale bar represents 20 μm. ∗*p* < 0.05. *E*, DiI-oxHDL uptake in peritoneal macrophages. Peritoneal macrophages were isolated from wildtype and CD36^−/−^ mice and were incubated with DiI-oxHDL (30 μg/ml). The scale bar represents 50 μm. ∗*p* < 0.05, compared with wildtype cells. *F* and *G*, the effects of oxHDL uptake in the presence of oxLDL (40 or 80 μg/ml). The RAW264.7 cells were pretreated with or without SSO followed by incubation with DiI-oxHDL and DiI-oxHDL + oxLDL. The values are presented as the mean ± SD from three independent experiments. ∗*p* < 0.05. DiI, 1,1′-diotadecyl-3,3,3′,3′-tetramethylindocarbocyanine perchlorate; HDL, high-density lipoprotein; ns, not significant; oxHDL, oxidized high-density lipoprotein.

To analyze the effects of oxHDL on lipid uptake in macrophages, the cells were exposed to 1,1′-diotadecyl-3,3,3′,3′-tetramethylindocarbocyanine perchlorate (DiI) fluorescence–labeled oxHDL, and the fluorescence intensity indicating the cellular oxHDL uptake level was detected by fluorescence microscopy. Intense and bright staining was evidently found in DiI-oxHDL–treated cells (Fig. 3*A*). However, DiI-HDL resulted in negligible or moderate intense and diffuse staining (Fig. 3*A*). Quantification analysis by flow cytometry showed that oxHDL endocytosis was increased more than three times compared with HDL uptake (Fig. 3, *B* and *C*). Given that enhanced lipid uptake leads to increased foam cell formation, we subsequently assessed the effects of oxHDL on intracellular lipid accumulation and foam cell formation by Oil Red O staining. As expected, oxHDL-treated macrophages had a notably increased lipid content, which presented more foam cells, as visualized by light microscopy, than the cells treated with HDL (Fig. 3*D*). To further explore the role of CD36 in oxHDL endocytosis, sulfosuccinimidyl oleate, a selective inhibitor of membrane CD36, was used to treat RAW264.7 and found that it caused a significant reduction in the oxHDL uptake and foam cell formation (Fig. 3, *A*–*D*). The results were further confirmed in CD36 KO cells, in which oxHDL uptake was dramatically lower in peritoneal macrophages from CD36^−/−^ mice than wildtype C57BL/6J mice (Fig. 3*E*). Moreover, we found that the uptake of oxHDL was reduced in the presence of oxLDL in RAW264.7 cells suggesting a competitive role of oxLDL in oxHDL uptake (Fig. 3*F*). Such effects were not observed in sulfosuccinimidyl oleate–treated macrophages (Fig. 3*G*). Together, our data demonstrated that oxHDL increases CD36-dependent lipid uptake.

### Inhibition of CD36 palmitoylation reduces CD36 lipid raft recruitment and oxHDL uptake

**Figure 4:**
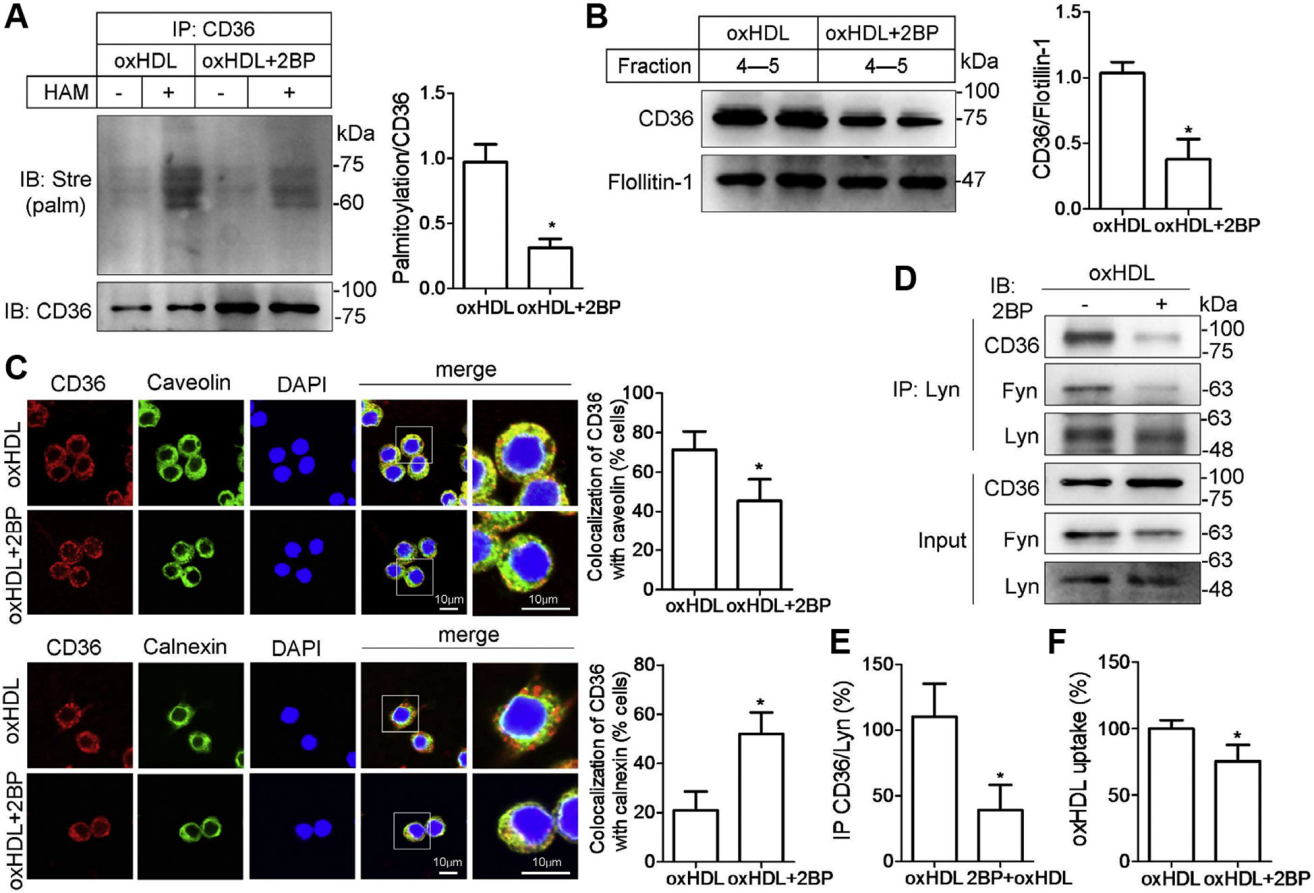
**The palmitoylation inhibitor 2-BP alters CD36 localization and ameliorates lipid uptake.***A*, 2-BP reduced oxHDL-induced CD36 palmitoylation. RAW264.7 cells were coincubated with oxHDL (80 μg/ml) and 2-BP (100 μM) for 24 h. CD36 palmitoylation was determined by ABE assay. *B*, 2-BP inhibited oxHDL-induced CD36 translocation to lipid rafts. Lysates from cells cotreated with oxHDL and 2-BP were fractionated by sucrose gradient ultracentrifugation. Lipid raft fractions 4 and 5 were collected, and CD36 protein levels were detected by immunoblotting. *C*, 2-BP reversed the effects of oxHDL on CD36 subcellular trafficking. RAW264.7 cells were prestimulated with oxHDL and treated with or without 2-BP in 24-well plates. The cells were fluorescently stained with CD36 (*red*), caveolin-1 (*green*), and DAPI (*blue*) (*upper panel*) and CD36 (*red*), calnexin (*green*), and DAPI (*blue*) (*lower panel*). The scale bars indicate 10 μm. Values are percentages of cells with colocalization in total cells. *D* and *E*, 2-BP reduced the assembly of CD36 with Lyn and Fyn. Lysates were coimmunoprecipitated with Lyn followed by immunoblotting for CD36, Fyn, and Lyn. *F*, 2-BP inhibited oxHDL uptake in RAW264.7 cells. Cells were pretreated with 2-BP for 24 h, followed by DiI-oxHDL (30 μg/ml) incubation for an additional 6 h. The mean fluorescence intensity was measured by flow cytometry. All results were repeated three times, and values were showed as mean ± SD. ∗*p* < 0.05, compared with oxHDL treatment. 2-BP, 2-bromopalmitate; ABE, acyl-biotin exchange; CD36, cluster of differentiation 36; DAPI, 4,6-diamidino-2-phenylindole; DiI, 1,1′-diotadecyl-3,3,3′,3′-tetramethylindocarbocyanine perchlorate; oxHDL, oxidized high-density lipoprotein.

To investigate whether CD36 palmitoylation modification is required by oxHDL-induced CD36 activity and lipid uptake, we blocked palmitoylation by using 2-bromopalmitate (2-BP), a known inhibitor of palmitoylacyl transferase (24). Macrophage RAW264.7 cells were coincubated with oxHDL and 2-BP or oxHDL alone, and the ABE assays revealed that CD36 palmitoylation was apparently detected in oxHDL-treated cells but remarkably inhibited by 2-BP (Fig. 4*A*). We next addressed the functional consequences of reduced CD36 palmitoylation. Detergent-resistant membranes recognized as lipid rafts were isolated by discontinuous sucrose density-gradient (5–40%) centrifugation. The 2-BP treatment significantly reduced CD36 recruitment into lipid rafts (Fig. 4*B*). The results from immunofluorescence assays showed that CD36 was punctately stained on the cell surface and colocalized with Cav-1 in oxHDL cells (Fig. 4*C*). The staining of CD36 and Cav-1 was separated by 2-BP, suggesting reduced expression of CD36 in the membrane (Fig. 4*C*). The colocalization of CD36 with calnexin was more frequently found in 2-BP-treated cells, suggesting that CD36 is detained in the ER caused by a lack of palmitoylation (Fig. 4*C*). We performed a coimmunoprecipitation assay to investigate the effects of depalmitoylation on the CD36/Lyn/Fyn association. The results showed that CD36 and Fyn were both less coimmunoprecipitated with Lyn in the presence of 2-BP, suggesting that the interaction among the three molecules was inhibited (Fig. 4, *D* and *E*). Finally, we tested whether oxHDL uptake was affected by palmitoylation blockade. Macrophages were coincubated with DiI-oxHDL and 2-BP and showed a significant decrease in the uptake of oxHDL measured by flow cytometry compared with only DiI-oxHDL treatment (Fig. 4*F*).

**Figure 5:**
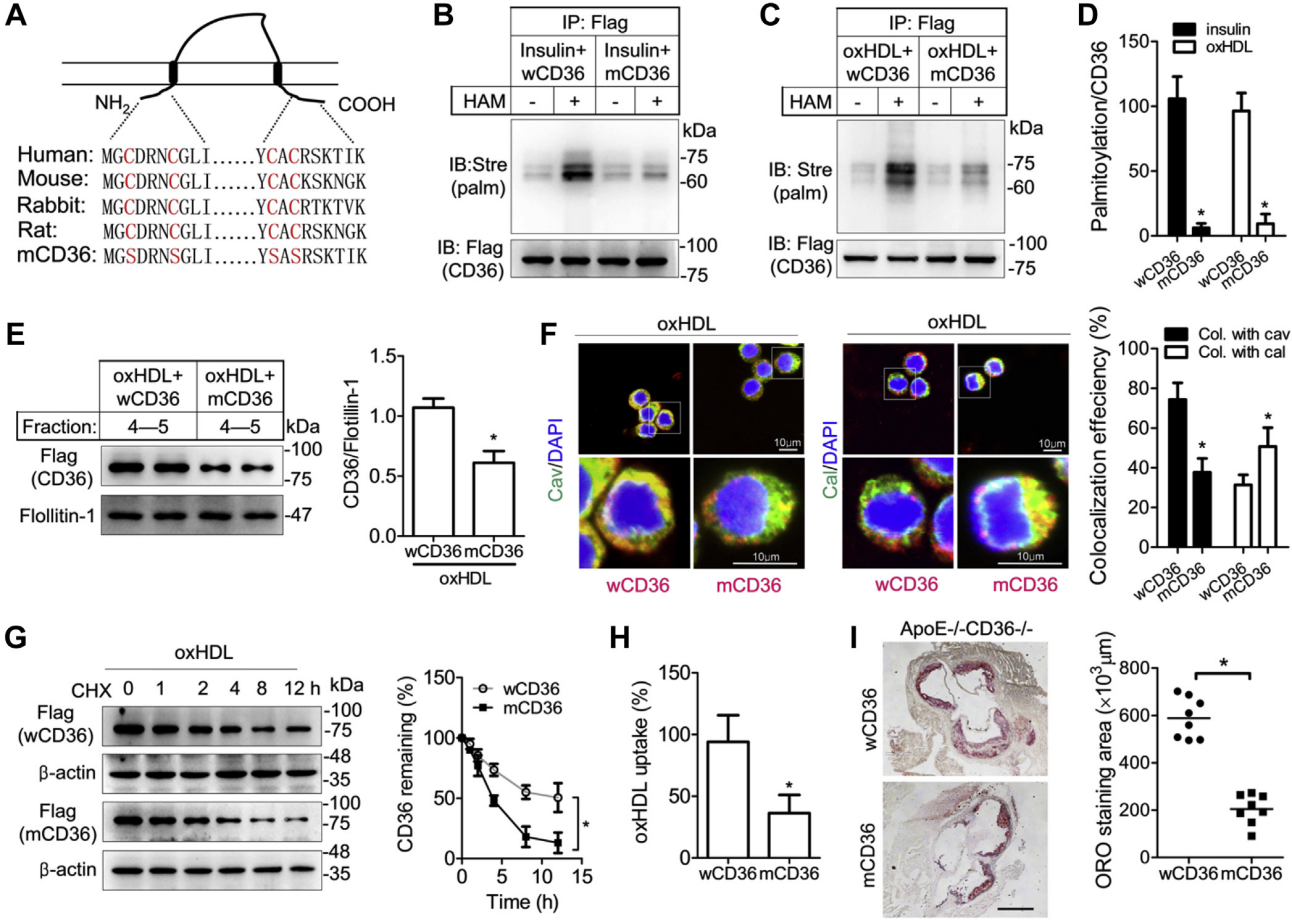
**Effects of CD36 palmitoylation disruption by direct point mutations in CD36 translocation and lipid uptake.***A*, schematics of the conserved structure of CD36 with two transmembrane domains, two short cytoplasmic domains, and a large extracellular loop in mammalian cells. Four conserved cysteine residues corresponding to palmitoylation (*red*) at the N and C termini were site-specifically mutated to serine in mutant CD36 (mCD36). *B*–*D*, cells were transfected with plasmids expressing wildtype CD36 (wC36) and mCD36 and then exposed to insulin or oxHDL. A reduction in CD36 palmitoylation was detected in mCD36-transfected cells. ∗*p* < 0.05, compared with wCD36. Data were expressed as mean ± SD (n = 3). *E*, disruption of CD36 palmitoylation by mutation reduced lipid raft recruitment in cells exposed to oxHDL. Cells were transfected with wCD36 and mCD36 following oxHDL incubation. Lipid rafts were isolated by sucrose gradient ultracentrifugation, and fractions 4 and 5 were collected for immunoblotting. Values are expressed as CD36/flotillin-1 relative to wCD36-transfected cells. ∗*p* < 0.05, compared with wCD36 (mean ± SD, n = 3). *F*, confocal images of CD36 subcellular translocation. Cells transfected with wCD36 or mCD36 were incubated with oxHDL and costained with CD36 (*red*), caveolin-1 (*green*), and DAPI (*blue*) (*left*) and CD36 (*red*), calnexin (*green*), and DAPI (*blue*) (*right*). The scale bars represent 10 μm. Values are percentages of cells with colocalization in total cells. ∗*p* < 0.05, compared with wCD36 (mean ± SD, n = 3). *G*, CD36 degradation assay. Cells were transfected with wCD36 or mCD36 and then incubated with oxHDL for an additional 24 h. Protein stability was analyzed by cycloheximide (CHX) chase assay within 12 h. Data were calculated as a percentage of total protein without CHX addition. ∗*p* < 0.05. Data were mean ± SD (n = 3). *H*, oxHDL uptake in cells transfected with mCD36. After transfection with wCD36 or mCD36, the cells were incubated with DiI-oxHDL for an additional 6 h. Lipid uptake indicated by fluorescence intensity was measured by flow cytometry. ∗*p* < 0.05, compared with wCD36. Data were mean ± SD (n = 3). *I*, lipid accumulation in mouse aorta. Double KO ApoE^−/−^CD36^−/−^ mice were infected with lentivirus-mediated vectors encoding wCD36 or mCD36. Frozen sections at the aortic root were stained with Oil Red O. The scale bar represents 250 μm. The graph represents the quantification of the area positive for lipid staining. Data are the average value of six sections per mouse (mean ± SD, n = 8 mice/group). ∗*p* < 0.05. CD36, cluster of differentiation 36; DAPI, 4,6-diamidino-2-phenylindole; DiI, ,1′-diotadecyl-3,3,3′,3′-tetramethylindocarbocyanine perchlorate; oxHDL, oxidized high-density lipoprotein.

Given that 2-BP is a general pharmacological palmitoylation blocker, we directly disrupted CD36 palmitoylation to investigate its role in oxHDL effects. We site-specifically mutated the CD36 cysteine residues, which undergo palmitoylation (cys3, cys7, cys464, and cys466) into serine (Fig. 5*A*). Mutant CD36 (mCD36) and wildtype CD36 (wCD36) were then introduced into RAW264.7 macrophages. To verify the performance of mCD36, the cells were induced by insulin, which has been well demonstrated to increase CD36 palmitoylation (13). The results from the ABE assay revealed that wCD36 was markedly palmitoylated (Fig. 5*B*). However, the palmitoylation was dramatically reduced in the cells transfected with mCD36, which suggests that postmodification was successfully inhibited by the mutations (Fig. 5, *B* and *D*). Next, we detected palmitoylation in the two kinds of transfected cells in the presence of oxHDL. Similar results were obtained, in which palmitoylation was evidently found in wCD36 but decreased in mCD36 (Fig. 5, *C* and *D*). Moreover, we further found that mCD36 membrane lipid raft assembly was reduced with palmitoylation blockade (Fig. 5*E*). The results were confirmed by immunofluorescence staining. In contrast to the colocalization of wCD36 and Cav-1, there was less mCD36 detected at the plasma membrane (Fig. 5*F*). Furthermore, mCD36 localization with calnexin was observed in more cells than wCD36, suggesting that CD36 trafficking to plasma is inhibited or delayed when the palmitoylation sites are displaced (Fig. 5*F*). In addition, we examined whether the CD36 stability induced by oxHDL is affected by changing the palmitoylation sites. Macrophages transfected with wCD36 and mCD36 were incubated with oxHDL. Results from the cycloheximide (CHX) chase assay showed that rapid degradation was detected in mCD36 at 4 h after CHX treatment, and the difference was more significant at several hours than in wCD36 (Fig. 5*G*). Measurement of oxHDL uptake in mCD36-transfected cells showed that cellular DiI-oxHDL was significantly reduced in comparison to wCD36-transfected cells (Fig. 5*H*). Finally, to further investigate the effects of CD36 depalmitoylation in mice, we infected CD36 and ApoE double KO (DKO) mice with lentivirus-mediated wCD36 or mCD36. After 12 weeks of high-fat diet (HFD) induction, cytosections at aortic roots were prepared and stained with Oil Red O. The DKO mice containing mCD36 showed significantly less lipid accumulation than the mice infected with wCD36, which accounted for a 65% reduction (588 × 10^3^ ± 30 × 10^3^ μm^2^ *versus* 204 × 10^3^ ± 22 × 10^3^ μm^2^) (Fig. 5*I*). The plasma triglycerides and total cholesterol levels did not significantly change between the two groups (Fig. S4). The *in vivo* data at least demonstrated that CD36 palmitoylation is involved in lipid uptake and foam cell formation in mice, although we cannot exclude the contribution of oxLDL, which is also elevated by HFD.

### DHHC6 contributes to oxHDL-induced CD36 palmitoylation

**Figure 6:**
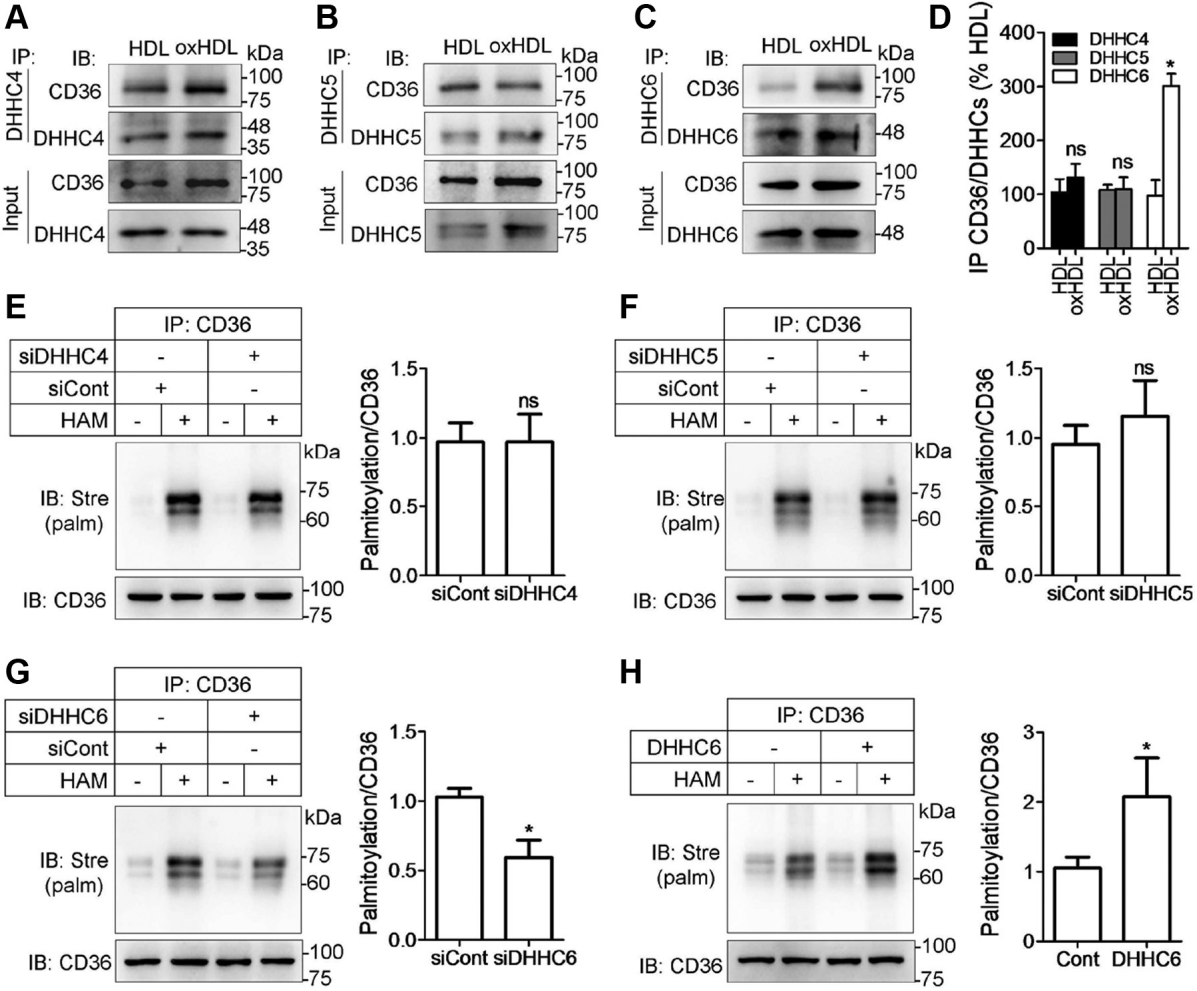
**DHHC6 interacts with CD36 and is required for CD36 palmitoylation.***A*–*D*, RAW264.7 cells were incubated with or without oxHDL. CD36 was immunoprecipitated with either DHHC4 (*A*), DHHC5 (*B*), or DHHC6 (*C*) antibodies. Blots were probed with CD36 and the DHHCs. ∗*p* < 0.05, compared with HDL. *E*–*G*, CD36 palmitoylation in cells after DHHC4, DHHC5, and DHHC6 knockdown. Cells were treated with siRNA specific to either DHHC4 (*E*), DHHC5 (*F*), or DHHC6 (*G*) and then exposed to oxHDL. The cell lysates were assayed by the ABE method. Scramble siRNAs were used as controls. ∗*p* < 0.05, compared with control siRNA. *H*, CD36 palmitoylation in DHHC6-overexpressing cells. Plasmids with the DHHC6 complete encoding sequence were transfected into RAW264.7 cells. Palmitoylated CD36 was detected by the ABE method, and the level was quantified. Relative values were expressed as mean ± SD from three independent experiments. ∗*p* < 0.05, compared with the control. ABE, acyl-biotin exchange; CD36, cluster of differentiation 36; DHHC, Asp-His-His-Cys; HDL, high-density lipoprotein; ns, not significant; oxHDL, oxidized high-density lipoprotein.

Having established that oxHDL increases CD36 palmitoylation and lipoprotein endocytosis, we attempted to explore the underlying mechanisms controlling the modification. In mammalian cells, there are at least 23 DHHC motif–containing enzymes known as PATS, which catalyze the S-palmitoylation reaction (25). Previous studies have reported that DHHC4, DHHC5, and DHHC6 are involved in the CD36 palmitoylation process (26, 27). To determine the potential CD36 palmitoylating enzymes, we performed a coimmunoprecipitation assay to investigate the interaction between CD36 and the individual PATs. The results showed a moderate increase in CD36/DHHC4 binding by oxHDL (Fig. 6, *A* and *D*). OxHDL caused the same or even slight reduction in the association of CD36 and DHHC5 (Fig. 6, *B* and *D*). However, DHHC6 coimmunoprecipitated more with CD36 in cells treated with oxHDL than HDL, and the association was stronger among the three DHHCs (Fig. 6, *A*–*D*). Next, we knocked down the three endogenous DHHCs by specific siRNAs in oxHDL-stimulated macrophages and found that CD36 palmitoylation was not notably changed by treatment with siRNA targeting DHHC4 or DHHC5 compared with scramble siRNA controls (Fig. 6, *E* and *F*). Silencing DHHC6 with a specific siRNA caused a 42% reduction in CD36 palmitoylation (Fig. 6*G*). The palmitoylated CD36 also increased significantly in macrophages overexpressing DHHC6 (Fig. 6*H*). Thus, the data suggest that the palmitoylation of CD36 is dependent on DHHC6 in response to oxHDL.

### Selenoprotein K is involved in oxHDL-induced CD36 palmitoylation

**Figure 7:**
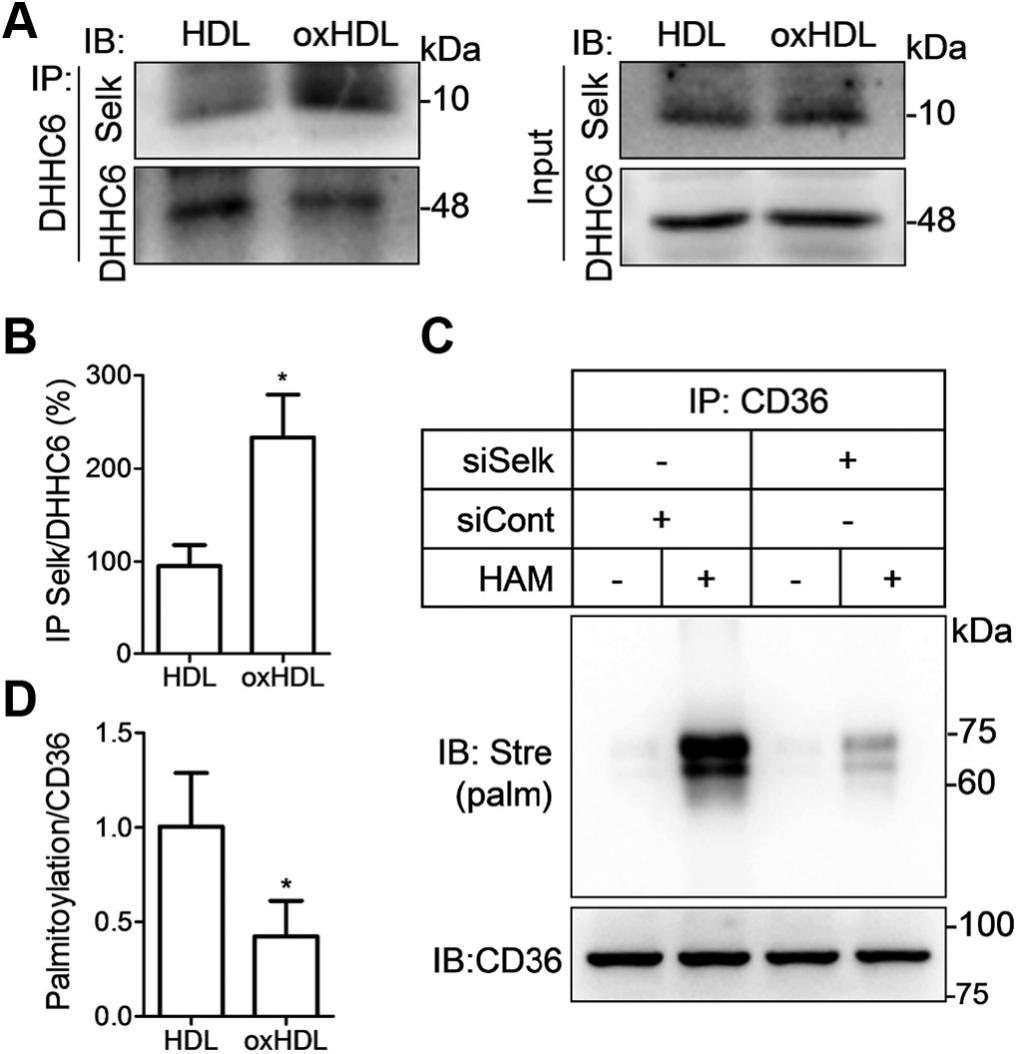
**SelK binding to DHHC6 facilitates oxHDL-induced CD36 palmitoylation.***A* and *B*, RAW264.7 macrophages were treated with HDL or oxHDL. The interaction between SelK and DHHC6 was detected by coimmunoprecipitation. *C* and *D*, ABE assay of CD36 palmitoylation. The cells were transfected with SelK siRNA or scramble siRNA and then subjected to oxHDL stimulation. ∗*p* < 0.05, compared with HDL (mean ± SD, n = 3). ABE, acyl-biotin exchange; CD36, cluster of differentiation 36; DHHC, Asp-His-His-Cys; oxHDL, oxidized high-density lipoprotein; SelK, selenoprotein K.

Selenoprotein K (SelK) is a selenium-containing protein that forms a complex with DHHC6 and enhances its acyl transferase activity at the ER membrane (28). We thus explored whether the effects of oxHDL on CD36 palmitoylation are mediated by SelK. The results revealed that more SelK was detected in DHHC6-precipitated lysates in oxHDL-treated cells compared with HDL, suggesting that oxHDL promotes the formation of the SelK–DHHC6 complex (Fig. 7, *A* and *B*). To further investigate the role of SelK in oxHDL-induced CD36 palmitoylation, we genetically disrupted SelK expression by siRNA. A lower level of CD36 palmitoylation was detected in the cells in the presence of siRNA specific for SelK compared with scrambled siRNA controls (Fig. 7, *C* and *D*). Therefore, data demonstrated that SelK, as a cofactor of DHHC6, facilitated oxHDL-induced CD36 palmitoylation.

### Cav-1 promotes CD36 membrane aggregation and oxHDL uptake

**Figure 8:**
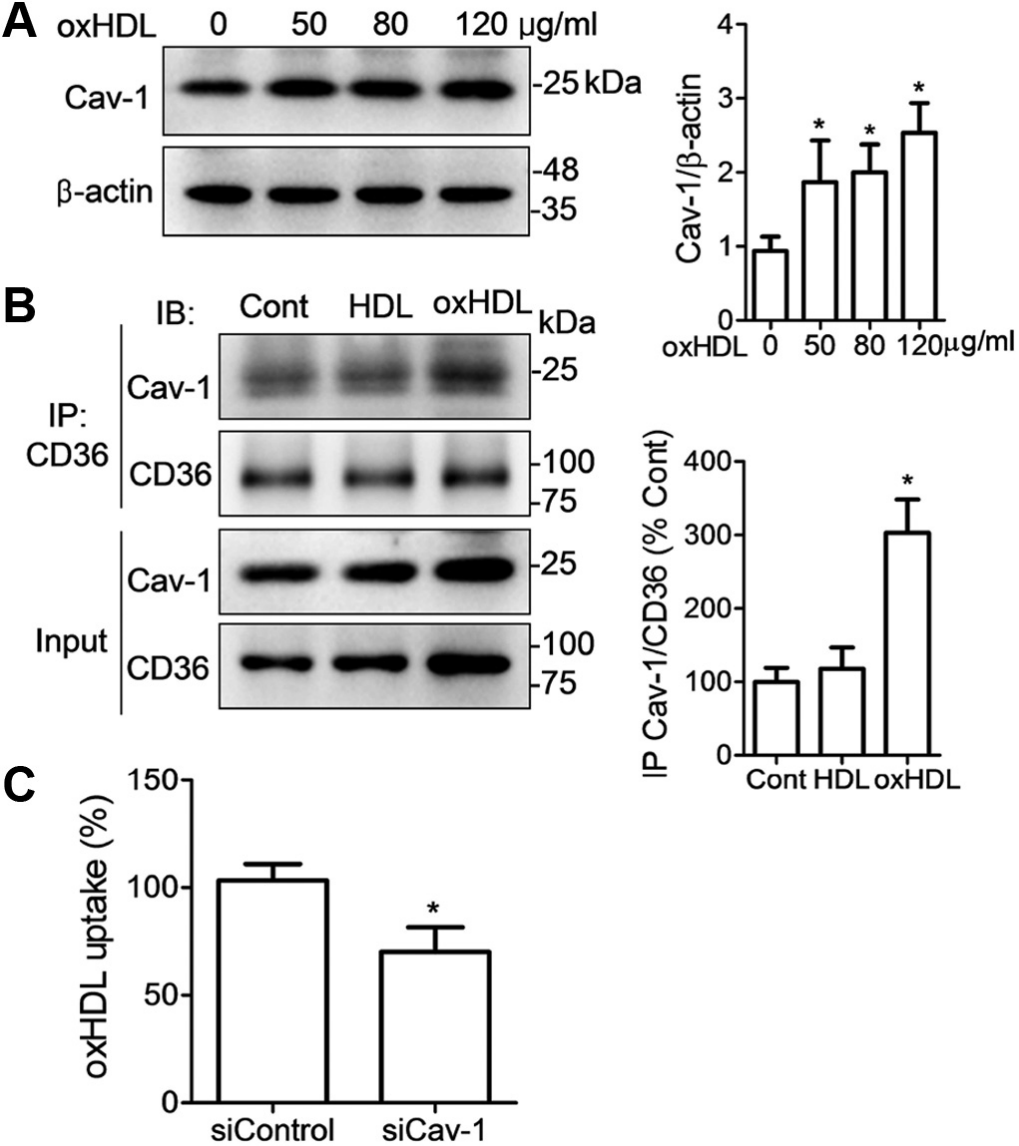
**Caveolin-1 (Cav-1) is involved in translocation of palmitoylated CD36 and oxHDL uptake.***A*, Cav-1 expression in oxHDL-incubated cells was detected by Western blotting at the indicated concentrations. *B*, coimmunoprecipitation analysis of Cav-1 and CD36 association in cells treated with oxHDL. *C*, oxHDL uptake measured by flow cytometer. RAW264.7 cells were transfected with Cav-1 siRNA for 72 h and incubated with DiI-oxHDL (30 μg/ml) for 6 h. All the data were from three independent experiments, and values were expressed as mean ± SD (n = 3). ∗*p* < 0.05, compared with untreated cells or control siRNA. CD36, cluster of differentiation 36; DiI, 1,1′-diotadecyl-3,3,3′,3′-tetramethylindocarbocyanine perchlorate; oxHDL, oxidized high-density lipoprotein.

Cav-1/CD36 interaction within lipid microdomains is coordinately involved in the uptake of FAs and lipoproteins (29, 30). However, a contradictory report demonstrated that the endocytosis of oxLDL mediated by CD36 is independent of Cav-1 (31). We then addressed the role of Cav-1 in oxHDL-stimulated lipoprotein uptake. The results showed that Cav-1 expression was significantly increased by oxHDL (Fig. 8*A*). More Cav-1 was coimmunoprecipitated by CD36 in oxHDL-treated cells than in native HDL-treated cells, suggesting that oxHDL increases Cav-1/CD36 association at the cell membrane (Fig. 8*B*). Furthermore, we found that uptake of DiI-oxHDL was reduced by 32% when Cav-1 was knocked down (Fig. 8*C*), which implies that Cav-1 functions in CD36-mediated oxHDL lipid uptake.

## Discussion

**Figure 9:**
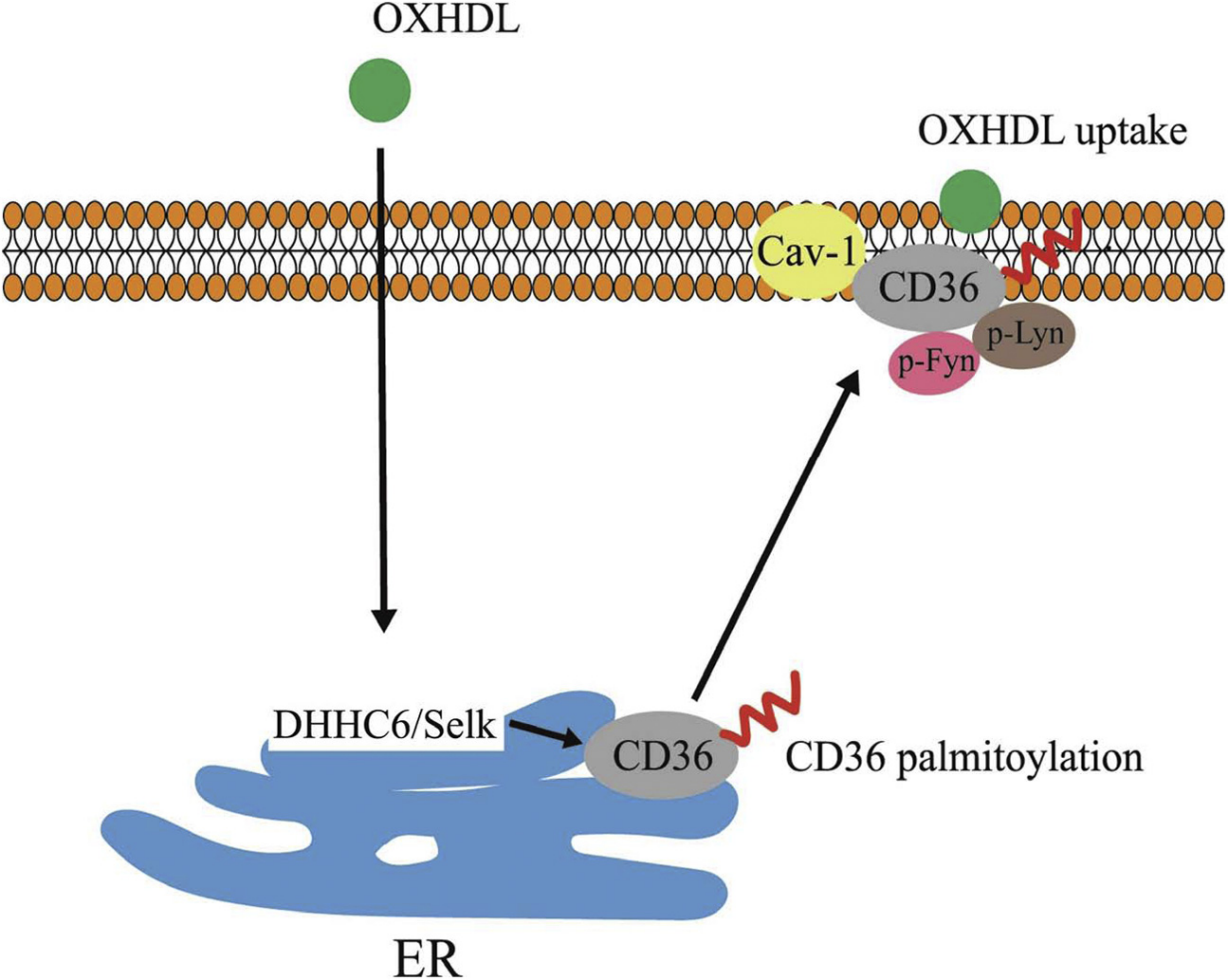
**Schematic diagram depicting the potential mechanism of oxHDL-induced CD36 palmitoylation and foam cell formation.** CD36, cluster of differentiation 36; oxHDL, oxidized high-density lipoprotein.

The present study provides evidence that oxHDL promotes CD36 palmitoylation and targets membrane lipid rafts. OxHDL further activates CD36 signaling by increasing CD36/Lyn/Fyn complex formation and downstream JNK activity in macrophages, which consequently increases oxHDL uptake and foam cell formation. We further demonstrated that the PAT DHHC6 and the cofactor Selk were required for CD36 modification, intracellular translocation, and oxHDL uptake (Fig. 9).

Palmitoylation of CD36 increases its cellular trafficking and cell surface localization in adipocytes, which facilitates oleate uptake (26). Inhibition of CD36 palmitoylation reduces its hydrophobicity and accessibility to the plasma membrane and causes a decrease in the binding and uptake of long-chain FAs, such as palmitate, in hepatic cells, which protects mice from nonalcoholic steatohepatitis and fibrosis (19). This evidence demonstrates that CD36 palmitoylation is deeply involved in FA metabolism and physiopathological function. CD36 is also a major SR that exhibits high affinity for lipoproteins both native and oxidative forms and mediates their internalization. Especially, the modified lipoproteins such as oxLDL and oxHDL are well known to stimulate oxidative stress and inflammation, resulting in multiphysiopathological consequences. Therefore, whether such oxidative lipoproteins influence CD36 palmitoylation and thus causes cellular physiopathological changes has not been well described. Here, we demonstrate that oxHDL elicits enhanced CD36 palmitoylation and efficient incorporation into lipid rafts, which is a major finding of this study. In addition, we found that the oxLDL also increases the CD36 palmitoylation (data not shown). However, the detailed mechanism might be different from those in oxHDL. DHHC5 showed a dramatic increase in association with CD36 in oxLDL-induced macrophages, while the binding of CD36/DHHC4 or CD36/DHHC6 was not significantly changed in cells responsive to oxLDL exposure (data not shown).

HDL, as a receptor of free cholesterol, maintains lipoprotein homeostasis by modulating cholesterol efflux from macrophages, thus promoting RCT, which renders HDL a protective role in atherogenesis. However, HDL is prone to oxidation by various reactive oxygen species, and oxHDL has proatherogenic properties. In this study, we proved that oxHDL, to some extent similar to oxLDL, enhances lipid uptake and foam cell formation, which implies that oxHDL might increase the lipid burden in vessels and trigger atherogenesis. Furthermore, we found that the proatherogenic effects of oxHDL were mechanistically mediated by increasing CD36 palmitoylation and activating downstream cascades, especially Lyn and Fyn. Therefore, our studies raise the possibility that interfering with CD36 post-translational modification might be a potential strategy for dealing with lipoprotein disorders and atherosclerosis.

CD36 has been demonstrated to be an initiator of the signaling cascade involved in Lyn and Fyn kinases to induce macrophage inflammation (21). Kennedy *et al.* (32) found that high cholesterol induced CD36-mediated JNK phosphorylation and cytokine release, thus promoting atherogenesis. CD36 deficiency results in reduced plasma inflammatory cytokines and chemokines and reduced macrophage infiltration in adipose tissue (33). It is well acknowledged that HDL acquires severe proinflammatory properties once it is oxidized and then directly contributes to atherosclerosis (3, 34). Therefore, apart from foam cell formation triggered by oxHDL that was demonstrated in this study, it is interesting to further explore whether the oxHDL-induced inflammatory response also occurs through the same CD36-mediated signaling cascade.

CD36 interacts with a diverse set of ligands. In addition to FAs and lipoproteins, CD36 has also been demonstrated to be a phagocytic pattern recognition receptor for pathogens (35, 36). By cooperation with Toll-like receptor 2/6, CD36 binds and internalizes *Staphylococcus aureus* as well as its cell wall component lipoteichoic acid in macrophages (35). Importantly, the effect is dependent on the COOH-terminal cytoplasmic domain of CD36, which initiates Toll-like receptor 2/6 signaling, leading to macrophage phagocytosis and cytokine production (35). CD36 also mediates the engulfment of various bacteria, such as *Escherichia coli*, *Klebsiella pneumoniae*, and *Salmonella typhimurium* by modulating the JNK signaling pathway (36). In addition, a study showed that CD36 functions as a phagocytic receptor for viruses, which enhances the hepatitis C virus attachment and replication within mammalian cells (37). This further supports the important role of CD36 in the innate immune response. We note that the palmitoylation of cell surface proteins such as nucleotide oligomerization domain (NOD)–like receptors 1 and 2 is required in immune response to bacteria (38). Our studies found that palmitoylated CD36 enabled macrophages to efficiently endocytose oxHDL, which thus provides a clue to explore whether the modification of CD36 is involved in the phagocytosis of pathogenic microbes.

To date, a total of 23 subtypes in the DHHC family that catalyze protein palmitoylation have been identified in mammals. A previous study showed that DHHC4 and DHHC5 are responsible for CD36 palmitoylation at different steps, in which DHHC4 palmitoylates CD36 at the Golgi apparatus and DHHC5 maintains modification at the cell surface in preadipocytes (26). In macrophages, impaired CD36 surface aggregation and defective palmitoylation were found in the absence of Selk, suggesting the important role of Selk in CD36 modification. Selk binds to DHHC6 through the SH3/SH3 binding domain and increases the catalytic efficiency by reducing hydrolyzation of the thioester and stabilizing the palmitoyl–DHHC6 intermediate (27). Therefore, we focused on DHHC4, DHHC5, and DHHC6 and performed coimmunoprecipitation and knockdown experiments to identify the specific enzyme controlling CD36 palmitoylation in this study. We found that Selk and DHHC6 were involved in oxHDL-stimulated CD36 palmitoylation, whereas DHHC4 and DHHC5 showed no such effects. The results suggest that different catalyst systems might exist in different cell types.

Each member of the DHHC family exhibits distinct subcellular localization. They are most frequently found in the ER, Golgi, and plasma membrane (39). DHHC4 and DHHC5 are distributed at the Golgi and membrane and undergo CD36 palmitoylation at different steps, as discussed previously (26, 39). DHHC6 resides at the ER membrane, where it palmitates substrate proteins such as the ER chaperone calnexin (40), ER E3 ligase gp78 (41), and inositol 1,4,5-triphosphate receptor (28). In this study, we observed that oxHDL increased CD36 plasma membrane trafficking while trapping CD36 in the ER when it blocked palmitoylation by 2-BP or mutation at the four cytoplasmic cysteine residues (Figs. 4 and 5) supporting the finding that CD36 palmitoylation was regulated by DHHC6 in the cell response to oxHDL stimulation, through we do not know how the oxHDL affects DHHC6 and subsequently the CD36 palmitoylation in the current study. We note that JNK forms a complex with DHHC17, and the direct interaction promotes neuronal injury (42). Considering that oxHDL could stimulate CD36 downstream molecules including JNK demonstrated in this study, the role of JNK or other mitogen-activated protein kinases such as extracellular signal–regulated kinase 1/2 and p38 in the oxHDL-activated DHHC6 is worth further investigation.

Because of the difficulty in finding a way to specifically induce oxHDL production in mice or recruit patients with spontaneously higher serum oxHDL, we could not verify the relationship between oxHDL and CD36 palmitoylation *in vivo* in this study. Otherwise, they would provide more insight and direct evidence for our better understanding of the causal role of oxHDL in CD36 post-translational modification, though we have demonstrated that CD36 palmitoylation contributed to aortic foam cell accumulation in mice (Fig. 5*H*).

In addition, there are still some questions remaining to be solved. OxHDL upregulated CD36 expression both at mRNA and protein levels (Fig. S3). However, we did not provide evidences of how the oxHDL induces CD36 expression and to what extent the increased expression of CD36 contributes to foam cell formation in this study, though we have demonstrated that the increased palmitoylation of CD36 in response to oxHDL stimulation promoted macrophage-derived foam cell formation. Another, given that scavenger receptor class B type I, as an important HDL receptor, is involved not only in cholesterol efflux from macrophages but also in liver selective uptake during RCT, it should not be ignored and remains interesting to explore the role of scavenger receptor class B type I when we investigate oxHDL uptake. Finally, HDL can be oxidized by various oxidants and enzymes. HDL is more often modified by hypochlorite and myeloperoxidase *in vivo* than CuSO_4_ used in this study (43, 44). Therefore, whether different sources of oxHDL bind to different receptors or confer other types of CD36 modifications needs further investigation.

In conclusion, we have shown that oxHDL promotes CD36 palmitoylation by DHHC6/SelK in macrophages. The post-translational modification enhances CD36 membrane lipid raft targeting and CD36 downstream signaling, which facilitates efficient oxHDL endocytosis, as summarized in Figure 9. This study reveals a central role of CD36 palmitoylation in oxHDL-induced lipid uptake and furthers our understanding of regulatory mechanisms in foam cell formation and atherogenesis.

## Experimental procedures

### Cell culture and treatments

Murine RAW264.7 macrophages were cultured in Dulbecco's modified Eagle's medium (HyClone) supplemented with 10% fetal bovine serum. Human THP-1 monocytes grew in complete RPMI1640 medium (HyClone) with 10% fetal bovine serum. Peritoneal macrophages were collected by intraperitoneal lavage with 8 ml Dulbecco's modified Eagle's medium at 4 days after an intraperitoneal injection of 1 ml 3% thioglycollate solution from 8-week-old male C57BL/6 mice. All cells were incubated in a humidified incubator at 37 °C with 5% CO_2_. Macrophages were treated with 80 μg/ml HDL or oxHDL (Yiyuan Biotechnology) for 24 h except for specific indications. Plasmids encoding FLAG-tagged wCD36 and mCD36 that replaced the four palmitoylation sites with serine (GenePharma) were transfected with Lipofectamine 2000 according to the manufacturer's instructions. (Invitrogen).

### HDL oxidation and oxHDL uptake assay

HDL was obtained from Yiyuan Biotechnologies. Oxidative modification was performed by dialysis against 200 μM CuSO_4_/PBS for 24 h at room temperature following dialysis in 200 μM EDTA and PBS for 24 h at 4 °C. OxHDL was labeled with fluorescent DiI and was added to the culture media at a final concentration of 30 μg/ml. After incubation for 6 h at 37 °C, the cells were washed with PBS three times and imaged by fluorescence microscopy (TE2000; Nikon). For the quantification assay of DiI-oxHDL uptake, the cells were harvested and resuspended in PBS and analyzed by flow cytometry (BD Biosciences). The mean fluorescence intensity of at least 10,000 events acquired for each sample was recorded.

### Immunofluorescence staining

RAW264.7 cells were seeded on coverslips at the bottom of a 24-well plate. After treatments, cells were fixed with 4% paraformaldehyde for 20 min and blocked with 5% bovine serum albumin for 30 min. The cells were then incubated with primary antibodies for 2 h at room temperature followed by the corresponding secondary FITC/rhodamine-conjugated antibody for 1 h at room temperature. The samples were then washed with PBS and mounted in antifade reagent containing 4,6-diamidino-2-phenylindole. Images were acquired using a fluorescence microscope (Nikon).

### siRNA knockdown

For RNA interference, siRNAs for DHHC4, DHHC5, and DHHC6 were synthesized from GenePharma Corporation; siRNAs for SelK and Cav-1 were obtained from Santa Cruz Biotechnology. RAW264.7 cells at 60% confluence in a 6-well plate were transfected with specific siRNA oligonucleotides (50 nM) using Lipofectamine RNAiMAX Transfection Reagent according to the manufacturer's instructions (Invitrogen). After 72 h, cells were harvested for further analysis.

### Animals and treatments

Mice were housed in 12 h light–dark conditions at 22 °C with free access to water and food. ApoE^−/−^CD36^−/−^ DKO mice were generated by crossing ApoE KO mice (ApoE^−/−^) (GemPharmatech) and CD36 KO mice (CD36^−/−^) (Cyagen). At least eight male DKO mice were infected with lentiviral-mediated wCD36 or mCD36 by tail vein injection. The mice were fed an HFD (21% fat and 0.15% cholesterol) for 12 weeks at 8 weeks old. After diet induction, animals were sacrificed by anesthesia with an overdose of pentobarbital sodium, and the hearts were collected. Serial cryostat sections (8 μm thickness) at the aortic root were prepared and stained with 0.3% Oil Red O for 15 min. Lipid accumulation of the lesion was determined by measuring the positive staining area within the total cross-sectional vessel. The experiments adhered to guiding principles approved by the Ethical Committee for Animal Care and Use at the Guizhou Medical University.

### Western blotting

Cells were lysed in radioimmunoprecipitation assay (RIPA) buffer (50 mM Tris, pH 7.4, 150 mM NaCl, 1% Triton X-100, 1% sodium deoxycholate, 0.1% SDS, and protease inhibitor cocktail) (Beyotime Biotechnology). After determining the concentrations by bicinchoninic acid assay (Beyotime), proteins were mixed with SDS loading buffer and resolved on 10 to 12% SDS-PAGE. The samples were then transferred to polyvinylidene difluoride membranes (Millipore Corporation). After blocking with 5% (w/v) nonfat dry milk in Tris-buffered saline and 0.1% Tween-20 (pH 7.6) for 1 h at room temperature, the membranes were immunoblotted with the indicated antibodies overnight at 4 °C and subsequently probed with horseradish peroxidase–conjugated secondary antibodies for 1 h at room temperature. The blots were washed three times with Tris-buffered saline and 0.1% Tween-20 and detected by enhanced chemiluminescence (Beyotime) as described by the supplier.

### Immunoprecipitation

Cells were washed three times with PBS and lysed on ice in 1 ml RIPA lysis buffer (Beyotime). After centrifugation at 13,000*g* and 4 °C for 10 min to remove insoluble debris, the supernatant was quantified using the bicinchoninic acid method (Beyotime). Aliquots of lysates were then incubated with antibody for 3 h at 4 °C and precipitated with ProteinA/G Sepharose beads for another 2 h at 4 °C with rotation. After washing three times with lysis buffer, the precipitated proteins were eluted from the beads by boiling with SDS loading buffer for 5 min and subjected to Western blotting analysis.

### ABE analysis

Protein palmitoylation was determined by the ABE method (45). In brief, cells were lysed with cold RIPA lysis buffer containing 50 mM *N*-ethylmaleimide (NEM). Samples were immunoprecipitated by adding CD36 antibody overnight and Protein-A/G Sepharose beads for 3 h at 4 °C in a rotating shaker. The precipitate was blocked in lysis buffer with 10 mM NEM followed by washing to remove unbound NEM with stringent buffer (lysis buffer, 10 mM NEM, and 0.1% SDS) and lysis buffer. The sample was split into two equivalent samples: one was incubated with 1 M hydroxylamine (HAM) treatment (+HAM sample) for 50 min at room temperature to cleave thioester bonds at palmitoylated cysteines; another sample without HAM was used as a control (−HAM sample). After one wash in lysis buffer (pH 6.2), the beads were incubated with 500 μl lysis buffer containing 1 μM biotin-BMCC (Thermo Fisher Scientific) with rotation to selectively label the palmitoylated cysteines. The resulting thiol-biotinylated proteins were further detected with streptavidin–horseradish peroxidase (Beyotime) by Western blotting.

### Lipid raft isolation

Isolation of detergent-resistant lipid rafts was performed by discontinuous sucrose gradient ultracentrifugation. Cells were lysed in 2 ml ice-cold RIPA lysis buffer and homogenized with a 25-gauge needle. The lysates were added to 2 ml of 80% sucrose in morpholinoethane sulfonic acid–buffered saline containing 25 mM MES (pH 6.5) and 0.15 M NaCl to achieve 4 ml of 40% sucrose. The sample was then overlaid with 4 ml of 35% sucrose and 4 ml of 5% sucrose in morpholinoethane sulfonic acid–buffered saline buffer. After centrifugation at 200,000*g* for 16 h at 4 °C in an SW41 rotor (Beckman), 12 sucrose gradient fractions were collected from the top, and equal volumes of each fraction were run on SDS-PAGE followed by immunoblotting analysis. Flotillin-1 was used as lipid raft marker.

### CHX chase experiment

RAW264.7 macrophages were transfected with plasmids encoding wCD36 and CD36 mutants, both with FLAG tags, for 36 h. CHX (50 μg/ml) was added to inhibit *de novo* protein synthesis, and the cells were lysed as described previously at the indicated time points after CHX addition. Protein levels were analyzed by immunoblotting.

### Statistical analysis

Data are expressed as the mean ± SD from at least three independent experiments. Statistical significance was determined by Student's *t* test or one-way ANOVA followed by Tukey’s post hoc test. A value of *p* < 0.05 was considered statistically significant. All statistical analyses were performed with the GraphPad Prism 5.0 program (GraphPad Software, Inc).

## Data availability

All data are contained within the article and supporting information.

## Supporting information

This article contains supporting information.

## Conflict of interest

The authors declare that they have no conflicts of interest with the contents of this article.
