# Supplementary material for: Oxidized high-density lipoprotein promotes CD36 palmitoylation and increases lipid uptake in macrophages
Source: J Biol Chem. 2022 Apr 29;298(6):102000. doi: 10.1016/j.jbc.2022.102000 (PMC9144050; doi:10.1016/j.jbc.2022.102000)
Supplement: Supplemental Figures S1–S4 [file mmc1.doc]

**Supplementary figures and legends**

**Supplementary figure 1**

**Fig. S1.** oxHDL aggregation and the effects of oxHDL on cell viability. *A*, Lipoprotein turbidity was determined by measuring the absorbance at 450 nm of 100 μl of HDL (1.5 mg/ml) and oxHDL (1.5 mg/ml) in a microplate reader. **P* <0.05, compared with HDL. *B*, Cytotoxicity of oxHDL was detected by Cell Counting Kit-8 (CCK-8). Cells were incubated with the indicated doses of oxHDL for 24 h followed by addition with CCK-8 solution for another 1 hour. The cell viability was determined using a 450 nm absorbance measurement. **P*<0.05, compared with cells without treatment (mean±SD, n=3). *C*, Cell apoptosis induced by oxHDL (80 μg/ml or 120 μg/ml) for 24 h was measured by [TUNEL assay](http://www.baidu.com/baidu.php?url=K00000K3Zd4fCW_uE9YkL31_fQoXAtKBlbJgGEwtV9OUP8jCuLBI3OZXkRa4luMGUx9xLwdI4G4rkbAeRuE1hzzFUsuxuTNZUG2d_lEykCP2MV5LYfEXnjg3DmXAAX8pY5u-bbUmyAUKkZrGZm22H1gwJgsUQbqwVc03LwcecQDtoZflUH2L84x5GQXTSznrIZZHqJbjR8iYFsr1jGAzdPZrJfvA.Db_NR2Ar5Od663rj6thU3yI5FKUZLeRlrKYdYUZlZS1Sg1OgSoZfux7dsRP5QAeKPa-BqM76l32AM-YG8x6Y_f33X8a9G4pauVQZlZS1Sg1Og9zyIolZgKfYt_QrMAzONDkO3ZYAm3Sg3xUem3vIZO6xfW_o4x-S9zOhSZ-H7dsRP5Qal26h26kexOKzOCxe54x5qxQg3eVOWyGJIGHz3qis1f_IPheWbR0.U1Yk0ZDqN7NrwRs0TA-W5H00TZPGuv3qm1f1mWcYmhf1P19-PyDzP1m4mhN9uHmdmHb1PvRYuWn0IjdYIy4-UaRznAVGI0KGUHYznWR0u1dEuZCk0ZNG5yF9pywd0ZKGujYkn0KWpyfqnHD30AdY5HDsnH-xnH0kPdtznjRkg1csPWFxn1msnfKopHYs0ZFY5HR4rfKBpHYkPH9xnW0Yg1RsnsKVm1Ykrjmzrj0drjTkg1D4nH04P104rj7xnW0dnNtznHmYrHn1rH0dPdts0Z7spyfqn0Kkmv-b5H00ThIYmyTqn0K9mWYsg100ugFM5H00TZ0qPWfsPWm1nW6s0A4vTjYsQW0snj0snj0s0AdYTjYs0AwbUL0qn0KzpWYs0Aw-IWdsmsKhIjYs0ZKC5H00ULnqn0KBI1Y30A4Y5H00TLCq0A71gv-bm1dsTzdMXh410A-bm1dcHbD0TA9YXHY0IA7zuvNY5Hnkg1nkP7tzP6KYIgnqnHfvPjm4njc4nHn1PjRdPWnsrfKzug7Y5HDvn1bkrHfdnHb3P1R0Tv-b5HbLrA79uy7-nj0snjfsPhD0mLPV5Rf4rHbYfYPjnjIKPWndrDm0mynqnfKsUWYs0Z7VIjYs0Z7VT1Ys0ZGY5H00UyPxuMFEUHYsg1Kxn7tsg100uA78IyF-gLK_my4GuZnqn7tsg1Kxn7ts0ZK9I7qhUA7M5H00uAPGujYs0ANYpyfqQHD0mgPsmvnqn0KdTA-8mvnqn0KkUymqn0KhmLNY5H00pgPWUjYs0A7buhk9u1Yk0Akhm1Ys0AwWmvfq0Zwzmyw-5Hm1njcsP6KBuA-b5HuAnYNjnW6LrHbdrjD3PDPDrjTkrRc1wHRvPj63rj9D0AqW5HD0mMfqnfKEmgwL5H00ULfqn0KETMKY5H0WnanWnansc10Wna3snj0snj0Wnansc10WQinsQW0snj0snankQW0snj0sn0K3TLwd5HT1PjbsPHD0TNqv5H08rjKxna3sn7tsQW0sg108nj7xna3sP-tsQWbLg108PjFxn0KBTdqsThqbpyfqn0KzUv-hUA7M5H00mLmq0A-1gvPsmHYs0APs5H00ugPY5H00mLFW5HDvnjT3&us=newvui&xst=TjYvPj0vPWnzrj0KmWYvwWP7f1c3P1b4PH6krjwjwj6LnH-anYRdPWf3rj63w0715HDsrHD4P1nzn103rjmsP1fLrj6zg1czPNts0gTqIZN8uys6pv-Y0gDqIZN8uysKIHYLn1f4njRk0gfqnHm1rHD4PjRzn07VTHYs0W0aQf7Wpjdhmdqsms7_IHYs0yP85yF9pywdpAqVuNqsusDvPHmvPW64rHc&cegduid=P1nYrH0dnf&solutionId=6627330&word=&ck=8582.14.1639194533653.0.0.492.169.0&shh=www.baidu.com&sht=baiduhome_pg&wd=). Scale bar, 20 μm. **P*<0.05, compared with oxHDL (80 μg/ml) (mean±SD, n=3).

**Supplementary figure 2**

**Fig. S2.** Effects of oxHDL on CD36 subcellular translocation in macrophages. *A,* Imaging analysis of CD36 localization in the cell membrane. RAW264.7 cells were incubated with oxHDL (80 μg/ml) for 24 h, and co-stained with CD36 antibody (red), cell membrane marker caveolin-1 antibody (green) and DAPI (blue). Images were aquired by confocal microscopy. *B,* Confocal images for CD36 localization in ER. Cells treated with oxHDL (80 μg/ml) for 24 h were stained with CD36 antibody (red), ER marker calnexin antibody (green) and DAPI (blue). Scale bars indicate 10 μm. The immunofluorescence images are representative of three independent experiments. **P*<0.05 (mean±SD, n=3), ns: not significant.

**Supplementary figure 3**

**Fig. S3.**  The expression of CD36, Fyn and Lyn in RAW264.7 macrophages. *A,B*, The cells were incubated with oxHDL or oxHDL + mβCD. The CD36 mRNA and protein levels were measured by qRT-PCR and Western blot, respectively. **P*<0.05, ns: not significant. *C, D*, The qRT-PCR analysis of Fyn and Lyn mRNA expression in cells treated with HDL or oxHDL. Data are the mean ± SD of three independent experiments. **P*<0.05, compared with HDL treated cells.

**Supplementary figure 4**

**Fig. S4.** Plasma triglycerides and total cholesterol levels were measured by enzymatic method. ApoE-/-CD36-/-mice were introduced with wildtype CD36 (wCD36) and mutant CD36 (mCD36) and subjected to high fat diet for 12 weeks. Data are expressed as mean ± SD, n=6 mice/group. ns: not significant, compared with double-knockout mice receiving wCD36.
